# Genetic Analyses Confirm SNPs in *HSPA8* and *ERBB2* are Associated with Milk Protein Concentration in Chinese Holstein Cattle

**DOI:** 10.3390/genes10020104

**Published:** 2019-01-30

**Authors:** Cong Li, Miao Wang, Wentao Cai, Shuli Liu, Chenghao Zhou, Hongwei Yin, Dongxiao Sun, Shengli Zhang

**Affiliations:** 1 Shaanxi Key Laboratory of Molecular Biology for Agriculture, College of Animal Science and Technology, Northwest A&F University, Yangling, Shaanxi 712100, China; congl@nwafu.edu.cn (C.L.); wangmiao9254@gmail.com (M.W.); 2 Department of Animal Genetics and Breeding, College of Animal Science and Technology, Key Laboratory of Animal Genetics and Breeding of Ministry of Agriculture, National Engineering Laboratory for Animal Breeding, China Agricultural University, Beijing 100193, China; wtaocai@163.com (W.C.); shuliliu1991@cau.edu.cn (S.L.); reddsbhj3@163.com (C.Z.); yinhongwei@cau.edu.cn (H.Y.); sundx@cau.edu.cn (D.S.)

**Keywords:** candidate genes, milk protein traits, association analyses, haplotypes, dairy cows

## Abstract

Heat shock 70 kDa protein 8 (*HSPA8*) and erb-b2 receptor tyrosine kinase 2 (*ERBB2*) were the promising candidates for milk protein concentration in dairy cattle revealed through previous RNA sequencing (RNA-Seq) study. The objective of this post-RNA-Seq study was to confirm genetic effects of *HSPA8* and *ERBB2* on milk protein concentration in a large Chinese Holstein population and to evaluate the genetic effects of both genes on other milk production traits. There were 2 single-nucleotide polymorphisms (SNPs) identified for *HSPA8* and 11 SNPs for *ERBB2* by sequencing 17 unrelated Chinese Holstein sires. The SNP-rs136632043 in *HSPA8* had significant associations with all five milk production traits (*p* = 0.0086 to *p* < 0.0001), whereas SNP-rs132976221 was remarkably associated with three yield traits (*p* < 0.0001). Nine (ss1996900615, rs109017161, rs109122971, ss1996900614, rs110133654, rs109941438, rs110552983, rs133031530, and rs109763505) of 11 SNPs in *ERBB2* were significantly associated with milk protein percentage (*p* = 0.0177 to *p* < 0.0001). A 12 Kb haplotype block was formed in *ERBB2* and haplotype associations revealed similar effects on milk protein traits. Our findings confirmed the significant genetic effects of *HSPA8* and *ERBB2* on milk protein concentration and other milk production traits and SNP phenotypic variances above 1% may serve as genetic markers in dairy cattle breeding programs.

## 1. Introduction

Molecular selective breeding strategies have been widely applied in animal breeding to improve the important economic traits of livestock. Identification of key genes or causal variations for economic traits is prerequisite to perform molecular selective breeding strategies [1,2]. Milk protein concentration is an important index to evaluate the nutritional value in cow’s milk. However, limited key genes or variations for milk protein concentration were found in dairy cattle [3,4,5,6,7,8,9]. Our initial RNA sequencing (RNA-Seq) study revealed that heat shock 70 kDa protein 8 (*HSPA8*) and erb-b2 receptor tyrosine kinase 2 (*ERBB2*) were candidate genes affecting milk protein traits in dairy cows [10]. It was observed that *HSPA8* (Log_2_fold change = −1.13, *q*-value = 2.91 × 10^−2^) and *ERBB2* (Log_2_fold change = 1.00, *q*-value = 5.25 × 10^−3^) were significant differentially expressed in bovine mammary tissues of cows in high and low milk protein percentage comparisons [10].

*HSPA8* is located in BTA15 with a total length of 4426 bp, containing 9 exons and 8 introns, and encoding 650 amino acids. *HSPA8* is an important gene in the MAPK pathway [11], which has a positive effect on protein synthesis by increasing the stability of mRNA through phosphorylation of the AU-rich element-binding protein [12]. *HSPA8* is highly expressed in the lactating mammary gland [10,13], which has a role in regulating protein folding and processing in the endoplasmic reticulum [14], likely in support of active milk protein synthesis [13]. *ERBB2* encodes a receptor of tyrosine kinases [15] and is located in BTA19 with a total length of 24,682 bp, containing 27 exons and 26 introns, and encoding 1255 amino acids. *ERBB2* could activate PI3K signaling pathway by directly binding of PI3K regulatory subunit p85 to phosphorylated tyrosine residues, which is known to regulate milk protein synthesis [16]. Most importantly, *HSPA8* is known to be downstream of *ERBB2* in human [17,18], indicating the probably regulatory effect of *ERBB2* to *HSPA8* and working together in specific biological processes.

Genetic analyses between selected genes and bovine milk production traits can provide valuable molecular information for the genetic improvement program for milk quality of dairy cattle. Therefore, the aims of this study were to identify genetic polymorphisms in *HSPA8* and *ERBB2*, to explore the linkages among single-nucleotide polymorphisms (SNPs), and to conduct the genetic effects analyses in a large Chinese Holstein population.

## 2. Materials and Methods

### 2.1. Animal Population and Phenotypic Data

A total of 1027 Chinese Holstein cows from 17 sire families were used to construct the study population. Family size ranges from 25 to 187 daughters with an average of 60 daughters per sire (Table S1). Cows were selected from 17 dairy farms in the Beijing Sanyuan Lvhe Dairy Farm Center, where regular and standard performance testing (Dairy Herd Improvement, DHI) have been implemented since 1999. Five milk production traits (305 d milk yield, 305 d protein yield, 305 d fat yield, average 305 d protein percentage, and average 305 d fat percentage) were collected from individual animal via the complete DHI data and were used for the subsequent analyses.

### 2.2. Genomic DNA Extraction

Blood samples were collected from 1027 Chinese Holstein cows via coccygeal vein and stored at −20 °C. The tubule frozen semen samples of 17 sires were collected from Beijing Bull Station. Genomic DNA was extracted from blood samples with a TIANamp Blood DNA kit (TIANGEN Biotech, Beijing, China) and from the semen samples using a standard phenol-chloroform procedure [19]. The quantity and quality of isolated DNA were confirmed before further analysis.

### 2.3. SNP Identification and Genotyping

A DNA pool was constructed from aforementioned 17 Holstein bulls (50 ng/uL of each individual) to identify potential SNPs that were involved in *HSPA8* and *ERBB2* genes. A total of 13 and 26 pairs of primers (Table S2) were designed to amplify all exons and their partial flanking intronic sequences based on the reference sequences of the bovine *HSPA8* (NCBI Reference Sequence: AC_000172.1) and *ERBB2* (NCBI Reference Sequence: AC_000176.1) referring to Bos_taurus_UMD_3.1 assembly using Primer3 web Program v.0.4.0 (http://primer3.ut.ee), respectively.

The polymerase chain reaction (PCR) was performed to amplify the pooled DNA from 17 sires with a final reaction volume of 25 μL, comprising of 50 ng genomic DNA, 0.5 μL of each primer, 2.5 μL 10× PCR buffer, 2.5 mM each of dNTP, and 1 U of Taq DNA polymerase (Takara Biotechnology Co., Ltd., Dalian, China). The PCR protocol was 5 min at 94 °C for initial denaturing, followed by 34 cycles at 94 °C for 30 s, 56 °C for 30 s, 72 °C for 30 s, and a final extension at 72 °C for 7 min. The amplification products were visualized by gel electrophoresis on 2% agarose gels, followed by photography under UV light. After that, 40 μL of each PCR product from the pooled DNA was bi-directionally sequenced using the ABI3730XL (Applied Biosystems, Foster City, CA, USA), and the sequences were aligned to the bovine reference sequences (UMD3.1) using BLAST (http://blast.ncbi.nlm.nih.gov/Blast.cgi) to identify potential SNPs. The subsequent genotyping analysis of the 1027 Chinese Holstein cows were performed with matrix-assisted laser desorption/ionization time of flight mass spectrometry (MALDI-TOF MS, Squenom MassARRAY, Bioyong Technologies Inc. HK) assay.

### 2.4. Linkage Disequilibrium Analysis

Pair-wise linkage disequilibrium (LD) was measured for each pair of SNPs genotyped within the *HSPA8* and *ERBB2* genes based on the criterion of D prime (D′) using Haploview [20]. Genotypes were firstly imputed for each individual using the Beagle3.2 software program [21]. Briefly, an iterative algorithm was applied for fitting a haplotype Hidden Markov Model (HMM) to genotype data that alternated between model building and sampling. In the model-building step, current estimates of phased haplotypes are used for building a new haplotype HMM, in the sampling step, new haplotypes are sampled for each individual conditional upon the genotype data and current haplotype HMM. Estimated phased haplotypes for the initial iteration are obtained by imputing missing genotypes at random according to allele frequencies and randomly phasing heterozygous genotypes. Accordingly, haplotype blocks where SNPs are in high LD (D′ > 0.90) were determined based on confidence intervals methods [22]. A haplotype with a frequency >5% was considered as a distinguishable haplotype, while the haplotypes with relative frequency <5% were pooled into a single group. Haplotype blocks within relative SNPs were applied to subsequent analyses to detect their associations with phenotypes.

### 2.5. Association and Haplotype Analyses

Hardy–Weinberg equilibrium test was conducted on each identified SNP. Chi-square was used to compare the number of expected and observed genotypes with a significance level of 0.05. The genetic effects of each candidate SNP or haplotype on five milk production traits were analyzed with the mixed procedure of SAS (SAS Institute Inc., Cary, NC, USA) with the following statistical model:

$$y_{\mathrm{ijklmn}}=\mu +F_{i}+YS_{j}+\;P_{k}+b\;\times M+G_{l}+\alpha_{m}+e_{\mathrm{ijklmn}}$$

where, $y_{\mathrm{ijklmn}}$ was the phenotypic value of each trait of cows (*n* = 1027 for each trait); *μ* was the overall mean; $F_{i}$ was the fixed effect of farm; $YS_{j}$ was the fixed effect of year-season; $P_{k}$ was the fixed effect of parity; M was the covariate effect of calving month; b was the regression coefficient of M; $G_{l}$ was the fixed effect corresponding to the genotype of polymorphisms or haplotype; $\alpha_{m}$ was the random polygenic effect, distributed as N (0, Aσ_a_^2^), with the additive genetic relationship matrix A and the additive genetic variance σ_a_^2^; and $e_{\mathrm{ijklmn}}$ was the random residual, distributed as N (0, Iσ_e_^2^), with identity matrix I and residual error variance σ_e_^2^. The overall reliability of the whole model was symbolized as ‘Goodness of Fit’, and was calculated by $R^{2}$ as following formula,

$$R^{2}=SS_{R}/SS_{T}=1-\;SS_{E}/SS_{T}=1-\;\sum \left({Residual}^{2}\right)/\sum {\left(Y-\bar{Y}\right)}^{2}$$

where, $R^{2}$ was the overall reliability; $SS_{R}$ was sum of squares of variables; $SS_{T}$ was total sum of squares; $SS_{E}$ was residual sum of squares; Residual was the residuals; Y was phenotypes of traits; $\bar{Y}$ was mean values of phenotypes of traits. The results showed that the $R^{2}$ of milk yield, milk protein percentage, milk fat percentage, milk protein yield and milk fat yield are 0.55, 0.50, 0.66, 0.73, 0.76, respectively, suggests that the model provide a good fit.

The differences among the effects of single SNPs or haplotypes on each trait were compared with Bonferroni correction. The significant level of the multiple tests was equal to the raw *P* value divided by number of tests. The additive (a), dominance (d) and allele substitution (α) effects were estimated according to the equation proposed by Falconer & Mackay [23], i.e., a=(AA−BB)/2, d=AB−(AA+BB)/2 and α=a+d(q−p), where AA and BB represent the two homozygous genotypes, AB is heterozygous genotype, and p and q are the allele frequencies of corresponding loci.

### 2.6. Phenotypic Variance

The proportion of phenotypic variance of the trait explained by a SNP was symbolized to show the effect of a SNP on a specific trait. The calculation formula is:

$$\mathrm{Phenotypic}\;\mathrm{variance}\;\mathrm{ratio}=2p\left(1-p\right)\alpha^{2}/\sigma_{p}^{2}$$

where p is the allele frequency of SNP, α is the average effect of gene substitution calculated by the linear mixed model, and $\sigma_{p}^{2}$ is the estimate of the phenotypic variance using the complete DHI data of Chinese dairy cattle population.

### 2.7. Ethics Approval and Consent to Participate

All protocols for collection of the blood and frozen semen samples of experimental individuals were approved by the Institutional Animal Care and Use Committee (IACUC) at China Agricultural University (Permit Number: DK996). We obtained written agreements from the cattle owners to use the samples and data.

## 3. Results

### 3.1. SNPs Identification

Two SNPs of rs136632043 and rs132976221 were identified for *HSPA8* gene, with one located in the 3′ regulatory region (3′-UTR) and the other located in the intron (Table 1). A total of 11 SNPs was discovered for *ERBB2* gene. Among these identified SNPs, eight SNPs (ss1996900615, rs109122971, ss1996900614, rs110133654, rs109941438, rs110552983, rs133031530 and rs109763505) were found within introns, one (rs133724008) was in the 5’ regulatory region (5′-UTR), and two SNPs (rs110735562 and rs109017161) were synonymous substitutions located in exons. All 13 identified SNPs of *HSPA8* and *ERBB2* genes were in Hardy–Weinberg equilibrium (*p* > 0.05, Table 2).

### 3.2. Single Locus-Based Association Analyses

SNP-rs136632043 was highly associated with all five milk production traits (*p* = 0.0086 to *p* < 0.0001; Table 3). SNP-rs132976221 also showed strong associations with three yield traits (milk yield, fat yield, and protein yield, *p* < 0.0001). There were six significant pairs of SNP-trait explaining phenotypic variations with greater than 1%, a range from 1.70 to 5.13% (Table 3). In addition, SNPs in *HSPA8* also showed the corresponding significant additive, substitution, or dominant effects on target traits (Table S3).

Nine SNPs (ss1996900615, rs109017161, rs109122971, ss1996900614, rs110133654, rs109941438, rs110552983, rs133031530, and rs109763505) in *ERBB2* were significantly associated with milk protein percentage (*p* = 0.0177 to *p* < 0.0001; Table 3). SNPs of rs110735562 and rs133724008 had significant associations with three yield traits (milk yield, fat yield, and protein yield, *p* = 0.0290 to *p* < 0.0001), whereas SNP of rs133724008 was also significantly associated with milk protein percentage (*p* < 0.0001; Table 3). Phenotypic variations explained by the 11 SNPs in *ERBB2* with greater than 1% were existed in four significant pairs of SNP-trait, ranging from 1.49 to 2.05% (Table 3). Nine SNPs (ss1996900615, rs109017161, rs109122971, ss1996900614, rs110133654, rs109941438, rs110552983, rs133031530, and rs109763505) had significant dominant effects on milk protein percentages (Table S3). SNP-rs110735562 had significant dominant effects on three yield traits. SNP-rs133724008 showed significant additive and substitution effects on three milk yield traits and significant dominant effects on milk protein percentage (Table S3).

### 3.3. LD and Haplotypes Analyses

**Figure 1:**
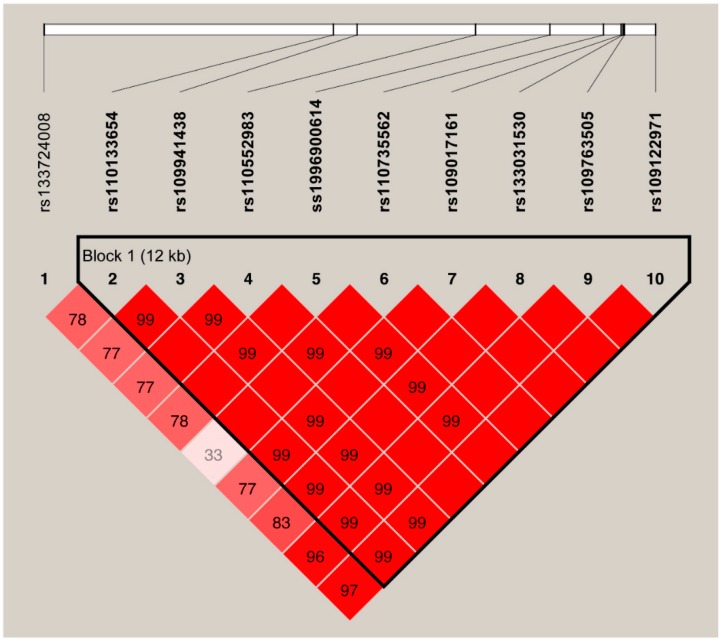
The haplotype blocks and pairwise linkage disequilibrium (LD) values (D′) for the ten SNPs in *ERBB2*. Legends: The values within boxes are pair wise SNP correlation (D′), bright red boxes without numbers indicate complete LD (D′ = 1). The brighter shade of red indicates higher LD.

One SNP (ss1996900615) identified for *ERBB2* gene was insertion/deletion (InDel), whereas the remaining 10 SNPs were used to perform LD analysis. Pair-wise D′ measures showed that nine SNPs in *ERBB2* were highly linked (D′ = 0.99~1.00) and one 12 Kb haplotype block comprising these nine SNPs were inferred (Figure 1), in which three main haplotypes were formed. The frequencies of the haplotypes ACGGGCTGC, GTCAATAAT, and GTCAGTAAT were 56.57%, 24.15%, and 15.04%, respectively (Table 4). Subsequently, haplotype-based analysis showed significant association of the haplotypes with all four milk production traits, except the milk yield (*p* = 0.0130 to *p* < 0.0001, Table 5). No LD was observed between the two identified SNPs for *HSPA8* gene.

## 4. Discussion

The results of the present study confirmed the significant genetic effects of *HSPA8* and *ERBB2* genes on milk protein traits in a large population of dairy cattle. The two candidate genes also had remarkable impacts on other milk production traits, which may provide new insights into the enhancement of milk profiles via selection strategies.

Two SNPs, rs110735562 and rs109017161 in exonic region of *ERBB2* showed significant associations with milk protein traits are synonymous variations, which could modify mRNA stability and further affect protein expression [24,25]. The SNPs have affect the promoter activity and gene expression [26], and a previous study reported that two SNPs (rs209535817 and rs210440016) in the 5′-UTR region of bovine *SAA2* (serum amyloid A2) gene impact the phenotype through altering the promoter activity [27]. Hence, it suggested that the genetic effect of SNP rs133724008 identified in the 5′-UTR of the *ERBB2* gene on milk production traits was likely due to the impacts on its transcription. Generally, mature miRNAs are bound to mRNA ribosomal complex and regulate the expression of target genes by complementary recognition of the 3′-UTR of the mRNA [28,29]. Thus, whether there are relative miRNAs binding to the 3′-UTR (Position: Chr15, 34216278-34216505) of *HSPA8* including identified SNPs were predicted by RNAhybrid software [30]. The results showed that miR-301a was targeted to the 3′-UTR including SNP rs136632043 (Position: Chr15, 34216487, Table S4), which indicated the significant associations of SNP rs136632043 in 3′-UTR of *HSPA8* with milk production traits probably resulted from miR-301a or unknown potential regulatory mechanism. Although an intron does not hold a sequence for coding protein, an important function of SNPs in introns in altering gene transcriptional level has been elucidated [31,32]. Additionally, the significant associations between SNPs in introns with milk protein traits are also likely due to their LD with true causative variation.

As a group of highly conserved and widely expressed proteins, heat-shock proteins (HSPs) play important physiological functions [33]. For example, HSPA8 has been validated as an evolutionarily conserved protein in swine and bovine [34]. HSPA8 is highly involved in many biological processes, including proteasomal degradation [35], catalyzing protein folding and clathrin uncoating [36], and other protein networks involved in protein catabolism, protein homeostasis, ubiquitination, carbohydrate metabolism and cell cycle control [37]. ERBB2 is a member of a family of transmembrane receptor tyrosine kinases involved in the regulation of cellular processes by modulation of several pathways, such as mTOR, MAPK, and PI3K/AKT pathways [38,39,40]. In consistent with the present study, the functional role of *ERBB2* on milk production traits has been also identified as positional candidate gene for lactation persistency in Canadian Holstein cattle [41]. Therefore, the results of the current study and previously published research indicate that *HSPA8* and *ERBB2* are promising candidate genes that have strong genetic effects on milk production traits. In addition, both single SNP-based and haplotype-based association analyses also suggest that the novel SNPs in these two genes may be used as potential genetic markers for genetic improvement in dairy breeding schemes.

Generally, a small proportion with less than 1% of the phenotypic variance was explained by polymorphisms underlying complex traits in livestock animals [42]. In the present study, six and four significant pairs of SNP-trait explaining phenotypic variations with greater than 1% were found in *HSPA8* and *ERBB2*, respectively. These results suggest that the subset of these large-effect SNPs (rs132976221, rs136632043, rs133724008, and rs110735562) could be used as potential genetic markers for further marker-assisted selection (MAS) in milk production traits, especially for milk protein traits. All identified SNPs in *HSPA8* and *ERBB2* genes could be incorporated into the SNP panels for genomic selection of dairy cattle breeding schemes and could be used to improve frequencies of the genetic markers that are positively related to milk production traits of interest.

## Figures and Tables

**Table 1:** Information for the identified single-nucleotide polymorphisms (SNPs) in *HSPA8* and *ERBB2* genes.

CHR	RefSNP	SNP Locus	Alleles	Location	Position	Gene
15	rs132976221	g.1585A>C	A/C	Intron-3	34219120	*HSPA8*
15	rs136632043	g.4218T>G	T/G	3′-UTR	34216487	*HSPA8*
19	rs133724008	g.873T>C	T/C	5′-UTR	40720963	*ERBB2*
19	ss1996900615	g.20982del	TC/.	Intron-19	40742818	*ERBB2*
19	rs110735562	g.21561A>G	A/G	Exon-21	40743397	*ERBB2*
19	rs109017161	g.22268T>C	T/C	Exon-23	40744104	*ERBB2*
19	rs109122971	g.23650T>C	T/C	Intron-26	40745486	*ERBB2*
19	ss1996900614	g.19414A>G	A/G	Intron-14	40741250	*ERBB2*
19	rs110133654	g.10727A>G	A/G	Intron-7	40732563	*ERBB2*
19	rs109941438	g.11680C>T	C/T	Intron-8	40733516	*ERBB2*
19	rs110552983	g.16431C>G	C/G	Intron-14	40738267	*ERBB2*
19	rs133031530	g.22346A>T	A/T	Intron-23	40744182	*ERBB2*
19	rs109763505	g.22400A>G	A/G	Intron-23	40744236	*ERBB2*

**Table 2:** Genotypic and allelic frequencies and Hardy–Weinberg equilibrium test of SNPs of *HSPA8* and *ERBB2* genes in Chinese Holstein cattle.

Gene	Position	Locus	Genotypes	N	Frequency	Allele	Frequency	Hardy–Weinberg Equilibrium χ^2^ Test
*HSPA8*	Intron-3	rs132976221 g.1585A>C	CA	432	0.428	A	0.702	*p* > 0.05
			AA	493	0.488	C	0.298	
			CC	85	0.084			
*HSPA8*	3′ prime UTR (exon9)	rs136632043 g.4218T>G	GT	277	0.274	G	0.175	*p* > 0.05
			GG	38	0.038	T	0.825	
			TT	696	0.688			
*ERBB2*	5′ flanking region	rs133724008 g.873T>C	CT	412	0.411	C	0.324	*p > 0.05*
			CC	119	0.119	T	0.676	
			TT	471	0.470			
*ERBB2*	Intron-19	ss1996900615 g.20982del	DEL.TC	471	0.469	DEL	0.604	*p* > 0.05
			DEL	372	0.370	TC	0.396	
			TC	162	0.161			
*ERBB2*	Exon-21	rs110735562 g.21561A>G	AG	362	0.358	A	0.243	*p* > 0.05
			AA	65	0.064	G	0.757	
			GG	585	0.578			
*ERBB2*	Exon-23	rs109017161 g.22268T>C	CT	458	0.458	C	0.607	*p* > 0.05
			CC	378	0.378	T	0.393	
			TT	164	0.164			
*ERBB2*	Intron-26	rs109122971 g.23650T>C	CT	473	0.473	C	0.568	*p* > 0.05
			CC	332	0.332	T	0.432	
			TT	196	0.196			
*ERBB2*	Intron-14	ss1996900614 g.19414A>G	AG	470	0.463	A	0.394	*p* > 0.05
			AA	165	0.162	G	0.606	
			GG	381	0.375			
*ERBB2*	Intron-7	rs110133654 g.10727A>G	AG	470	0.468	A	0.604	*p* > 0.05
			AA	372	0.370	G	0.396	
			GG	163	0.162			
*ERBB2*	Intron-8	rs109941438 g.11680C>T	CT	452	0.455	C	0.605	*p* > 0.05
			CC	375	0.378	T	0.395	
			TT	166	0.167			
*ERBB2*	Intron-14	rs110552983 g.16431C>G	CG	472	0.464	C	0.393	*p* > 0.05
			CC	164	0.161	G	0.607	
			GG	381	0.375			
*ERBB2*	Intron-23	rs133031530 g.22346A>T	AT	445	0.451	A	0.390	*p* > 0.05
			AA	162	0.164	T	0.610	
			TT	379	0.384			
*ERBB2*	Intron-23	rs109763505 g.22400A>G	AG	483	0.478	A	0.432	*p* > 0.05
			AA	195	0.193	G	0.568	
			GG	332	0.329			

**Table 3:** Associations of identified SNPs of *HSPA8* and *ERBB2* genes with milk production traits in Chinese Holstein cattle (Least square mean ± Standard error, LSM ± SE).

Locus	Genotypes	Milk Yield	Fat Yield	Fat Percentage	Protein Yield	Protein Percentage
*HSPA8* rs132976221 g.1585A>C	AA(493)	10307 ± 61.79 ^A^	361.58 ± 2.58 ^A^	3.572 ± 0.025	323.23 ± 1.88 ^A^	3.169 ± 0.009
	AC(432)	10639 ± 62.63 ^B^	373.69 ± 2.62 ^B^	3.580 ± 0.025	333.02 ± 1.91 ^B^	3.164 ± 0.009
	CC(85)	10465 ± 100.55 ^AB^	367.58 ± 4.24 ^AB^	3.576 ± 0.041	330.38 ± 3.09 ^B^	3.180 ± 0.014
	*p*-value	**<0.0001**	**<0.0001**	0.9436	**<0.0001**	0.4773
	Variance	**4.76 × 10^−2^**	**3.74 × 10^−2^**	1.48 × 10^−4^	**5.13 × 10^−2^**	8.83 × 10^−5^
*HSPA8* rs136632043 g.4218T>G	GG(38)	10632 ± 140.30 ^A^	403.60 ± 5.93 ^A^	3.797 ± 0.057 ^A^	338.32 ± 4.32 ^A^	3.192 ± 0.020 ^A^
	GT(277)	10339 ± 67.43 ^B^	363.93 ± 2.82 ^B^	3.586 ± 0.027 ^B^	325.44 ± 2.06 ^B^	3.188 ± 0.010 ^A^
	TT(696)	10532 ± 59.56 ^A^	364.61 ± 2.48 ^B^	3.536 ± 0.024 ^B^	328.70 ± 1.81 ^B^	3.154 ± 0.008 ^B^
	*p*-value	**0.0022**	**<0.0001**	**<0.0001**	**0.0086**	**<0.0001**
	Variance	1.28 × 10^−3^	**4.16 × 10^−2^**	**3.25 × 10^−2^**	1.46 × 10^−3^	**1.70 × 10^−2^**
*ERBB2* rs133724008 g.873T>C	CC(119)	10311 ± 90.86 ^a^	361.80 ± 3.81 ^a^	3.574 ± 0.037	324.73 ± 2.78 ^A^	3.195 ± 0.013 ^A^
	CT(412)	10469 ± 62.32 ^ab^	365.97 ± 2.60 ^ab^	3.542 ± 0.025	327.50 ± 1.89 ^A^	3.150 ± 0.009 ^B^
	TT(471)	10551 ± 62.29 ^b^	370.49 ± 2.60 ^b^	3.595 ± 0.025	331.81 ± 1.89 ^B^	3.178 ± 0.009 ^A^
	*p*-value	**0.0178**	**0.0290**	0.0666	**0.0069**	**<0.0001**
	Variance	**1.49 × 10^−2^**	9.02 × 10^−3^	9.40 × 10^−5^	9.89 × 10^−3^	**1.78 × 10^−2^**
*ERBB2* ss 1996900615 g.20982del	DEL(372)	10453 ± 63.86	366.24 ± 2.66	3.594 ± 0.026	327.94 ± 1.94	3.178 ± 0.009 ^a^
	DEL.TC(471)	10478 ± 61.61	367.51 ± 2.57	3.561 ± 0.025	328.49 ± 1.87	3.158 ± 0.009 ^b^
	TC(162)	10449 ± 83.25	366.96 ± 3.49	3.561 ± 0.033	328.21 ± 2.54	3.181 ± 0.012 ^a^
	*p*-value	0.8756	0.8715	0.3157	0.9526	**0.0140**
	Variance	5.87 × 10^−5^	1.58 × 10^−5^	9.68 × 10^−4^	2.45 × 10^−6^	1.60 × 10^−3^
*ERBB2* rs110735562 g.21561A>G	AA(65)	10305 ± 115.13 ^A^	364.26 ± 4.86 ^AB^	3.592 ± 0.046	323.00 ± 3.54 ^A^	3.180 ± 0.016
	AG(362)	10566 ± 66.63 ^B^	372.41 ± 2.78 ^A^	3.577 ± 0.027	331.66 ± 2.02 ^B^	3.171 ± 0.009
	GG(585)	10410 ± 58.40 ^A^	363.35 ± 2.43 ^B^	3.565 ± 0.023	325.84 ± 1.77 ^A^	3.165 ± 0.008
	*p*-value	**0.0060**	**0.0006**	0.7432	**0.0010**	0.4977
	Variance	**1.79 × 10^−2^**	6.48 × 10^−3^	9.48 × 10^−4^	**2.05 × 10^−2^**	2.37 × 10^−3^
*ERBB2* rs109017161 g.22268T>C	CC(164)	10465 ± 63.62	367.53 ± 2.66	3.599 ± 0.026	328.83 ± 1.94	3.180 ± 0.009 ^A^
	CT(458)	10465 ± 61.90	367.96 ± 2.59	3.569 ± 0.025	327.77 ± 1.89	3.157 ± 0.009 ^B^
	TT(378)	10422 ± 83.31	363.93 ± 3.50	3.549 ± 0.034	326.39 ± 2.55	3.178 ± 0.012 ^AB^
	*p*-value	0.8382	0.4394	0.2187	0.5892	**0.0062**
	Variance	6.05 × 10^−4^	2.78 × 10^−3^	3.24 × 10^−3^	1.58 × 10^−3^	5.71 × 10^−4^
*ERBB2* rs109122971 g.23650T>C	CC(332)	10457 ± 66.19	367.99 ± 2.77	3.608 ± 0.027	328.41 ± 2.02	3.179 ± 0.009 ^A^
	CT(473)	10488 ± 61.60	368.74 ± 2.57	3.563 ± 0.025	328.80 ± 1.87	3.157 ± 0.009 ^B^
	TT(196)	10377 ± 77.66	363.34 ± 3.26	3.564 ± 0.031	325.82 ± 2.37	3.185 ± 0.011 ^A^
	*p*-value	0.2843	0.1835	0.1368	0.3740	**0.0038**
	Variance	2.32 × 10^−3^	4.14 × 10^−3^	2.09 × 10^−3^	2.41 × 10^−3^	1.86 × 10^−3^
*ERBB2* ss 1996900614 g.19414A>G	AA(165)	10381 ± 82.85	363.42 ± 3.48	3.565 ± 0.033	325.46 ± 2.54	3.181 ± 0.012 ^A^
	AG(470)	10502 ± 61.53	368.89 ± 2.57	3.563 ± 0.025	329.13 ± 1.87	3.157 ± 0.009 ^B^
	GG(381)	10476 ± 63.60	368.21 ± 2.66	3.598 ± 0.026	329.29 ± 1.94	3.178 ± 0.009 ^A^
	*p*-value	0.2745	0.2179	0.2796	0.2308	**0.0078**
	Variance	3.64 × 10^−3^	4.98 × 10^−3^	8.93 × 10^−4^	5.28 × 10^−3^	1.75 × 10^−3^
*ERBB2* rs110133654 g.10727A>G	AA(372)	10501 ± 64.00	369.19 ± 2.68	3.592 ± 0.026	330.40 ± 1.95	3.178 ± 0.009 ^A^
	AG(470)	10490 ± 61.54	366.60 ± 2.56	3.548 ± 0.025	328.63 ± 1.87	3.155 ± 0.009 ^B^
	GG(163)	10416 ± 83.71	364.56 ± 3.52	3.549 ± 0.034	327.67 ± 2.56	3.180 ± 0.012 ^A^
	*p*-value	0.5360	0.3295	0.1291	0.4442	**0.0049**
	Variance	2.20 × 10^−3^	2.73 × 10^−3^	1.57 × 10^−3^	1.67 × 10^−3^	1.62 × 10^−3^
*ERBB2* rs109941438 g.11680C>T	CC(375)	10464 ± 63.85	367.66 ± 2.67	3.593 ± 0.026	329.19 ± 1.94	3.181 ± 0.009 ^A^
	CT(452)	10482 ± 61.95	367.23 ± 2.58	3.553 ± 0.025	328.40 ± 1.88	3.157 ± 0.009 ^B^
	TT(166)	10364 ± 82.72	360.55 ± 3.47	3.543 ± 0.033	324.45 ± 2.53	3.183 ± 0.012 ^A^
	*p*-value	0.2884	0.0724	0.1341	0.1331	**0.0034**
	Variance	3.78 × 10^−3^	9.51 × 10^−3^	2.76 × 10^−3^	7.37 × 10^−3^	1.57 × 10^−3^
*ERBB2* rs110552983 g.16431C>G	CC(164)	10440 ± 82.87	366.09 ± 3.48	3.559 ± 0.033	327.50 ± 2.53	3.177 ± 0.012 ^AB^
	CG(472)	10521 ± 61.62	368.52 ± 2.58	3.551 ± 0.025	330.20 ± 1.88	3.156 ± 0.009 ^A^
	GG(381)	10511 ± 63.46	370.25 ± 2.65	3.598 ± 0.026	331.32 ± 1.93	3.179 ± 0.009 ^B^
	*p*-value	0.5463	0.4421	0.1045	0.2855	**0.0080**
	Variance	1.90 × 10^−3^	2.49 × 10^−3^	1.07 × 10^−3^	4.35 × 10^−3^	5.06 × 10^−4^
*ERBB2* rs133031530 g.22346A>T	AA(162)	10420 ± 84.37	363.01 ± 3.54	3.539 ± 0.034	327.77 ± 2.58	3.182 ± 0.012 ^A^
	AT(445)	10452 ± 64.28	364.58 ± 2.68	3.544 ± 0.026	327.28 ± 1.95	3.155 ± 0.009 ^B^
	TT(379)	10457 ± 65.19	365.70 ± 2.72	3.587 ± 0.026	329.02 ± 1.98	3.181 ± 0.009 ^A^
	*p*-value	0.8889	0.7097	0.1197	0.6089	**0.0015**
	Variance	4.20 × 10^−4^	1.04 × 10^−3^	2.21 × 10^−3^	1.45 × 10^−4^	1.60 × 10^−3^
*ERBB2* rs109763505 g.22400A>G	AA(195)	10476 ± 77.76	368.62 ± 3.26	3.567 ± 0.031	330.12 ± 2.38	3.182 ± 0.011 ^a^
	AG(483)	10515 ± 61.66	369.25 ± 2.57	3.563 ± 0.025	330.07 ± 1.87	3.160 ± 0.009 ^b^
	GG(332)	10495 ± 66.45	369.09 ± 2.78	3.606 ± 0.027	329.97 ± 2.03	3.180 ± 0.009 ^a^
	*p*-value	0.8460	0.9776	0.1747	0.9973	**0.0177**
	Variance	1.65 × 10^−4^	4.54 × 10^−5^	1.50 × 10^−3^	5.58 × 10^−6^	6.97 × 10^−4^

Note: *p*-value refers to the results of association analysis between each SNP and milk production traits. Different letter (small letters: *p* < 0.05; capital letters: *p* < 0.01) superscripts (adjusted value after correction for multiple testing) indicate significant differences among the genotypes.

**Table 4:** Main haplotypes and their frequencies observed in *ERBB2* gene.

*ERBB2* Haplotypes	SNP1 G > A	SNP2 T > C	SNP3 C > G	SNP4 A > G	SNP5 A > G	SNP6 T > C	SNP7 A > T	SNP8 A > G	SNP9 T > C	Frequency (%)
ACGGGCTGC	A	C	G	G	G	C	T	G	C	56.57
GTCAATAAT	G	T	C	A	A	T	A	A	T	24.15
GTCAGTAAT	G	T	C	A	G	T	A	A	T	15.04

Note: The Ref number of each SNP can be found in the haplotype Figure 1. SNP1 = rs110133654, SNP2 = rs109941438, SNP3 = rs110552983, SNP4 = ss1996900614, SNP5 = rs110735562, SNP6 = rs109017161, SNP7 = rs133031530, SNP8 = rs109763505, SNP9 = rs109122971.

**Table 5:** Haplotype associations of the nine SNPs in *ERBB2* with milk production traits in Chinese Holstein cattle (LSM ± SE).

*ERBB2* Haplotypes	Milk Yield	Fat Yield	Fat Percentage	Protein Yield	Protein Percentage
H1H1(337)	10464 ± 70.84	366.88 ± 2.97 ^AC^	3.605 ± 0.029 ^a^	327.87 ± 2.16 ^A^	3.178 ± 0.010 ^A^
H1H2(272)	10536 ± 76.34	371.71 ± 3.19 ^A^	3.603 ± 0.031 ^a^	330.23 ± 2.33 ^A^	3.174 ± 0.011 ^A^
H1H3(169)	10348 ± 82.03	355.82 ± 3.45 ^B^	3.507 ± 0.033 ^b^	320.29 ± 2.51 ^B^	3.130 ± 0.011 ^B^
H2H2(65)	10296 ± 117.42	362.97 ± 4.96 ^AB^	3.593 ± 0.047 ^ab^	322.42 ± 3.61 ^AB^	3.183 ± 0.016 ^A^
H2H3(82)	10416 ± 108.62	357.93 ± 4.57 ^BC^	3.521 ± 0.044 ^ab^	323.81 ± 3.33 ^AB^	3.183 ± 0.015 ^A^
H3H3(19)	10122 ± 203.08	360.35 ± 8.60 ^AB^	3.612 ± 0.082 ^ab^	319.76 ± 6.27 ^AB^	3.180 ± 0.028 ^AB^
*p*-value	0.0538	<0.0001	0.0130	0.0009	0.0001

Note: *p*-value refers to the results of association analysis between each haplotype and milk production traits. Different letter (small letters: *p* < 0.05; capital letters: *p* < 0.01) superscripts (adjusted value after correction for multiple testing) indicate significant differences among the haplotypes. H1 = ACGGGCTGC, H2 = GTCAATAAT, H3 = GTCAGTAAT.

## Supplementary Materials

The following are available online at http://www.mdpi.com/2073-4425/10/2/104/s1. Table S1: The distribution of daughters from 17 herds in 17 farms. Table S2: PCR primers information of *HSPA8* and *ERBB2* genes. Table S3: Additive, dominant and allele substitution effects of the SNPs associated with milk production traits of *HSPA8* and *ERBB2* in Chinese Holstein cattle. Table S4: The predicted miRNAs targeted to the 3′-UTR of *HSPA8* gene.
