# Supplementary material for: Genetic Analyses Confirm SNPs in HSPA8 and ERBB2 are Associated with Milk Protein Concentration in Chinese Holstein Cattle
Source: Genes (Basel). 2019 Jan 30;10(2):104. doi: 10.3390/genes10020104 (PMC6409942; doi:10.3390/genes10020104)
Supplement: Supplementary file 1 [file genes-10-00104-s001.pdf]

Table S1. The distribution of daughters from 17 herds in 17 farms

Herd No.	01	02	10	15	17	32	33	37	38	43	44	45	46	55	56	64	71	Daughters	No. herds
00113			1			4	3		9	14			8	4	5	3	12	63	10
01902				22	1	1				1	20				1			46	6
01906	5										15	4			1			25	4
01907				16		5	2		7				6					36	5
01916					17	1				23	9			10			23	83	6
01927			39			8		5						3	8			63	5
01929	8	1	3	64				6	5	3	2			10	10		5	117	11
01930		1		1						1					8		24	35	5
02129		2		14		1					2		10				5	34	6
02909		26		3				6	28									63	4
02910			9					8		4	1				9			31	5
02912	11			1		1	1				12			7	9	6	5	53	9
02919									7	3		26	11	2	4	2	4	59	8
03543		10			1			5	23									39	4
99095					9	5		27	43	15	8			21		41	18	187	9
99995											3		9			36		48	3
97314				1		21	17				1				2	3		45	6
Total	24	40	52	122	28	47	23	57	122	64	73	30	44	57	57	91	96	1027	17

Table S2. PCR primers information of *HSPA8* and *ERBB2* genes

Primer	Location	Primer sequences (5’-3’)	Product size (bp)	Annealing Temp (°C)
HSPA8-1	5’ flanking region	F-CCAGCTGCTTAACTTCCCAC<br>R-GTGTTCGGGGCCTTGTTAAG	503	59
HSPA8-2	5’ flanking region	F-CTTAACAAGGCCCCGAACAC<br>R-GTCCCCGCACTTTTCACTTT	573	59
HSPA8-3	5’ flanking region	F-AAAGTGAAAAGTGCGGGGAC<br>R-GACAAAATGGCGGCTAAGGT	553	59
HSPA8-4	5’ flanking region	F-TGGACAAGTTACCCTCCCAG<br>R-GGAACTTTCATTCTCGCCCC	531	59
HSPA8-5	Exon-1	F-GGGGCGAGAATGAAAGTTCC<br>R-AAGAATTGCCAGACACCACC	433	59
HSPA8-6	Exon-2	F-CTCCCGCGTGATTATTTCCG<br>R-CGCCTCACACTTAACAGCTT	437	59
HSPA8-7	Exon-3	F-TGAACCTGCATTGACAGCAT<br>R-GCATCTTTGGTAGCCTGACG	**532****	59
HSPA8-8	Exon-4/5	F-GCAGGAGGATGTGGAGGTTA<br>R-CGGACAGCCCTCTTGTTTTC	587	59
HSPA8-9	Exon-5	F-TGGAGCAGAAAGAAACGTGC<br>R-TTAGGCCTCGTAACTGCTGT	591	59
HSPA8-10	Exon-6	F-ACAGCAGTTACGAGGCCTAA<br>R-TAGGCAAGACTGATCGCTCA	537	59
HSPA8-11	Exon-7/8	F-CCCTGAGTTTTCCCCTCTGT<br>R-CTGCTTCTTGAACCATGCGT	502	59
HSPA8-12	Exon-8	F-CGCTAAATGGAGTTGCAGGG<br>R-GGTTACATTGGAGGTCTTGGG	502	59
HSPA8-13	Exon-9	F-GAGTGACCCAAGACCTCCAA<br>R-CCCAAACCCACTTAACTACCC	**597****	59
ERBB2-1	5’ flanking region	F-AGTTGGTGATGGACAGGGAG<br>R-GGGGTGGAGAAAGAAGGACA	595	59
ERBB2-2	5’ flanking region	F-AGGTTCAGTCACTTGCCCAA<br>R-GAGGTGGGGATGGAAGGAAA	411	59
ERBB2-3	5’ flanking region	F-CAGTAGGTGCCTCTGATCCC<br>R-CAAGTTTGGAGACGGACAGG	**546****	59
ERBB2-4	5’ flanking region	F-TCTCCAAACTTGTGCTGCTG<br>R-GGCAATTCCCAGCTTCACTT	565	59
ERBB2-5	Exon-1	F-GAAACGGGCGAAAATTGCTG<br>R-GCCCTCTTACCCCACTTTCT	685	59
ERBB2-6	Exon-2	F-ACCTCTTCCCTGATTTCCCC<br>R-TGCATTCCACCCAGAGATGT	493	59
ERBB2-7	Exon-3	F-ACATCATCTCGTCCAGGCAT<br>R-CAGCTCTCAACAACCAGCAG	553	59
ERBB2-8	Exon-4	F-GCTCAAAGATGCTGAAGGGG<br>R-GAGAGGTAGTGAGCGAGACC	558	59
ERBB2-9	Exon-5/6	F-ACTAACAGCACCCATCTCCC	594	59

		R-GACAACACTGGATGGGATGC		
ERBB2-10	Exon-7	F-GCATCCCATCCAGTGTTGTC<br>R-GAGCCAAGTCTTGAACGCAA	**441****	59
ERBB2-11	Exon-8	F-CTCAGGCATGTTGTAAGGGC<br>R-TCCCCACATTGACAGGAGAC	**404****	59
ERBB2-12	Exon-9	F-CCCCTGTGTCTCCTGTCAAT<br>R-TCCAGCCAGTCACATGATGT	522	59
ERBB2-13	Exon-10/11	F-TCGCTCAGTCCTGTTCAACT<br>R-GCTGCAAACTCCCATGGAAA	547	59
ERBB2-14	Exon-12	F-CGAGTTCTGCATGAGTGAGC<br>R-CATGCACAGGGAAGTCGATG	556	59
ERBB2-15	Exon-13/14	F-CAGCTATCCCGCCTCTCTAG<br>R-AGCCTCCTTCCACTCTCCTA	**596****	59
ERBB2-16	Exon-15	F-CAGAACAGGGGTAGCAGACA<br>R-AGGCAGAGATGTCCCACTTT	**414****	59
ERBB2-17	Exon-16	F-TACAGAGGAGATGGCCAAGC<br>R-ACATGACACCTAGCAGCAGT	406	59
ERBB2-18	Exon-17/18	F-TTCCAAATGCCAGTGACAGC<br>R-GGCCACTCCCAATCCCTTAT	603	59
ERBB2-19	Exon-19	F-ATAAGGGATTGGGAGTGGCC<br>R-GCACTGAGAGGGTATGGGAA	**524****	59
ERBB2-20	Exon-20/21	F-TTCCCATACCCTCTCAGTGC<br>R-TCACTCGGTTCGCTTGTCTA	**518****	59
ERBB2-21	Exon-22	F-TGACGAGACAGAGTACCACG<br>R-ATGGGGTAAAGTGGTGGGAG	509	59
ERBB2-22	Exon-23	F-CCCTTTGGCACTGCATCATT<br>R-CCCCAGTCTCGACAACACTA	**534****	59
ERBB2-23	Exon-24/25	F-TCATCAGAGGGCTTTGGACT<br>R-GAGGTATCCAACGACACCCA	640	59
ERBB2-24	Exon-26/27	F-AGGCCCCATTTTCCCTAAGA<br>R-TGACAACCCCATTCTTCCCA	**700****	59
ERBB2-25	Exon-27	F-TGGGAAGAATGGGGTTGTCA<br>R-GCCATTACCCACTCTCCACT	692	59
ERBB2-26	Exon-27	F-CCAGCCTGACTTCTTCCAGA<br>R-ACTCCCAGTTTCCCCTCAAC	534	59

Note: **SNPs are detected.

Table S3. Additive, dominant and allele substitution effects of the SNPs associated with milk production traits of *HSPA8* and *ERBB2* in Chinese Holstein cattle

Gene	Locus	Genetic effect	Milk yield	Fat yield	Fat percentage	Protein yield	Protein percentage
HSPA8	rs132976221 g.1585A>C	Additive	-79.39	-3.00	-0.0017	**-3.57***	-0.0052
		Dominant	**253.13****	**9.11****	0.0058	**6.22****	-0.0104
		Substitution	-243.66	-8.93	-0.0055	-7.62	0.0015
HSPA8	rs136632043 g.4218T>G	Additive	50.04	**19.50****	**0.1303****	**4.81***	**0.0190***
		Dominant	**-243.14****	**-20.17****	**-0.0808***	**-8.07****	0.0151
		Substitution	-48.16	**11.35****	**0.0977****	**1.55****	0.0251
ERBB2	rs133724008 g.873T>C	Additive	**-119.74****	**-4.35***	-0.0106	**-3.54****	0.0082
		Dominant	37.76	-0.18	-0.0421	-0.76	**-0.0361****
		Substitution	**-133.35****	**-4.28***	0.0043	**-3.27****	0.0208
ERBB2	ss 1996900615 g.20982del	Additive	2.28	-0.36	0.0165	-0.14	-0.0015
		Dominant	27.42	0.91	-0.0165	0.41	**-0.0216****
		Substitution	8.01	-0.17	0.0131	-0.05	-0.0060
ERBB2	rs110735562 g.21561A>G	Additive	-52.55	0.45	0.0139	-1.42	0.0076
		Dominant	**208.13****	**8.60****	-0.0017	**7.23****	-0.0014
		Substitution	-159.48	-3.96	0.0148	-5.14	0.0083
ERBB2	rs109017161 g.22268T>C	Additive	21.18	1.80	0.0251	1.22	0.0013
		Dominant	21.25	2.23	-0.0052	0.16	**-0.0226****
		Substitution	25.73	2.28	0.0240	1.25	-0.0036
ERBB2	rs109122971 g.23650T>C	Additive	40.18	2.33	0.0221	1.30	-0.0031
		Dominant	71.05	3.07	-0.0229	1.69	**-0.0244****
		Substitution	49.65	2.74	0.01899	1.52	-0.0064
ERBB2	ss 1996900614 g.19414A>G	Additive	-47.57	-2.40	-0.0165	-1.92	0.0014
		Dominant	73.02	3.08	-0.0183	1.75	**-0.0229****
		Substitution	-63.09	-3.05	-0.0126	-2.29	0.0063
ERBB2	rs110133654 g.10727A>G	Additive	42.32	2.31	0.0214	1.37	-0.0011
		Dominant	32.15	-0.27	-0.0228	-0.40	**-0.0239****
		Substitution	49.01	2.26	0.0166	1.29	-0.0060
ERBB2	rs109941438 g.11680C>T	Additive	49.99	3.55	0.0253	2.37	-0.0007
		Dominant	67.54	3.13	-0.0154	1.58	**-0.0249****
		Substitution	64.31	4.21	0.0221	2.70	-0.0059
ERBB2	rs110552983 g.16431C>G	Additive	-35.91	-2.08	-0.0196	-1.91	-0.0013
		Dominant	45.30	0.35	-0.0276	0.79	**-0.0217****
		Substitution	-45.58	-2.16	-0.0138	-2.08	0.0034
ERBB2	rs133031530 g.22346A>T	Additive	-18.60	-1.35	-0.0241	-0.63	0.0002
		Dominant	12.93	0.23	-0.0193	-1.11	**-0.0265****
		Substitution	-21.47	-1.39	-0.0198	-0.38	0.0060
ERBB2	rs109763505 g.22400A>G	Additive	-9.28	-0.23	-0.0193	0.08	0.0011
		Dominant	29.36	0.40	-0.0235	0.03	**-0.0206****
		Substitution	-13.26	-0.29	-0.0161	0.07	0.0039

Note: The asterisk (*) means the additive, dominant or allele substitution effect of the locus indicate differ at P<0.05 and the asterisk (**) means the additive, dominant or allele substitution effect of the locus indicate differ at P<0.01.

Table S4 The predicted miRNAs targeted to the 3’-UTR of *HSPA8* gene

Start position	Length of miRNA targeted	Chr	Genome start	Genome end	Predicted miRNAs	MFE	Targeted
154	25	15	34216431	34216456	bta-miR-658	-32.4	no
152	22	15	34216429	34216451	bta-miR-1343-5p	-31.9	no
115	24	15	34216392	34216416	bta-miR-2291	-29	no
82	22	15	34216359	34216381	bta-miR-664a	-28.8	no
135	22	15	34216412	34216434	bta-miR-2442	-28.8	no
78	22	15	34216355	34216377	bta-miR-2418	-28.6	no
136	22	15	34216413	34216435	bta-miR-2407	-28.6	no
67	23	15	34216344	34216367	bta-miR-2438	-28.4	no
166	22	15	34216443	34216465	bta-miR-763	-28.3	no
79	22	15	34216356	34216378	bta-miR-669	-28.2	no
135	23	15	34216412	34216435	bta-miR-453	-27.7	no
72	23	15	34216349	34216372	bta-miR-2373-3p	-27.6	no
175	23	15	34216452	34216475	bta-miR-2453	-27.5	no
158	20	15	34216435	34216455	bta-miR-2897	-27	no
171	24	15	34216448	34216472	bta-miR-2344	-26.6	no
154	22	15	34216431	34216453	bta-let-7b	-26.4	no
40	22	15	34216317	34216339	bta-miR-449b	-26.1	no
136	22	15	34216413	34216435	bta-miR-34c	-26	no
174	23	15	34216451	34216474	bta-miR-671	-26	no
154	22	15	34216431	34216453	bta-miR-2413	-25.9	no
39	23	15	34216316	34216339	bta-miR-2486-5p	-25.8	no
135	22	15	34216412	34216434	bta-miR-21-3p	-25.8	no
151	23	15	34216428	34216451	bta-miR-2324	-25.8	no
166	22	15	34216443	34216465	bta-miR-2387	-25.8	no
185	20	15	34216462	34216482	bta-miR-2313-3p	-25.8	no
136	22	15	34216413	34216435	bta-miR-449a	-25.7	no
164	24	15	34216441	34216465	bta-miR-2443	-25.7	no
52	22	15	34216329	34216351	bta-miR-25	-25.6	no
106	22	15	34216383	34216405	bta-miR-2396	-25.6	no
178	22	15	34216455	34216477	bta-miR-2436-5p	-25.6	no
135	22	15	34216412	34216434	bta-miR-149-3p	-25.5	no
136	23	15	34216413	34216436	bta-miR-2467-3p	-25.5	no
51	22	15	34216328	34216350	bta-miR-92a	-25.2	no
67	24	15	34216344	34216368	bta-miR-574	-25.2	no
140	18	15	34216417	34216435	bta-miR-2899	-25.2	no
179	22	15	34216456	34216478	bta-miR-2474	-25.1	no
186	21	15	34216463	34216484	bta-miR-7860	-25.1	no
95	23	15	34216372	34216395	bta-miR-2330-5p	-25	no
138	23	15	34216415	34216438	bta-miR-2364	-25	no
183	24	15	34216460	34216484	bta-miR-2357	-25	no

40	21	15	34216317	34216338	bta-miR-2456	-24.9	no
12	22	15	34216289	34216311	bta-miR-34a	-24.8	no
50	23	15	34216327	34216350	bta-miR-367	-24.8	no
52	24	15	34216329	34216353	bta-miR-2327	-24.8	no
134	22	15	34216411	34216433	bta-miR-2447	-24.8	no
52	22	15	34216329	34216351	bta-miR-193a-5p	-24.6	no
66	21	15	34216343	34216364	bta-miR-33a	-24.6	no
104	22	15	34216381	34216403	bta-miR-2294	-24.6	no
159	22	15	34216436	34216458	bta-miR-1284	-24.6	no
167	21	15	34216444	34216465	bta-miR-1224	-24.6	no
177	23	15	34216454	34216477	bta-miR-2353	-24.6	no
65	23	15	34216342	34216365	bta-miR-32	-24.5	no
**186**	**25**	**15**	**34216463**	**34216488**	**bta-miR-301a**	**-24.5**	**yes**
39	23	15	34216316	34216339	bta-miR-181d	-24.4	no
54	23	15	34216331	34216354	bta-miR-4657	-24.3	no
84	20	15	34216361	34216381	bta-miR-2304	-24.3	no
88	22	15	34216365	34216387	bta-miR-2286	-24.3	no
135	21	15	34216412	34216433	bta-miR-2450a	-24.3	no
158	23	15	34216435	34216458	bta-miR-2285q	-24.3	no
135	21	15	34216412	34216433	bta-miR-2348	-24.2	no
155	22	15	34216432	34216454	bta-miR-2448-3p	-24.2	no
156	23	15	34216433	34216456	bta-miR-365-5p	-24.2	no
166	22	15	34216443	34216465	bta-miR-3432a	-24.2	no
54	25	15	34216331	34216356	bta-miR-301b	-24.1	no
77	20	15	34216354	34216374	bta-miR-449d	-24.1	no
85	22	15	34216362	34216384	bta-miR-206	-24	no

Note: Start Position: start position of base in 3’-UTR (34216278-34216505) of *HSPA8* targeted to miRNAs; **Length of miRNAs targeted:** the length of miRNAs targeted to 3’-UTR; **Chr:** Chromosome for *HSPA8* gene; **Genome Start:** start position of miRNAs targeting 3’-UTR of *HSPA8*; **Genome End:** end position of miRNAs targeting 3’-UTR of *HSPA8*; **MFE:** Minimum free energy, less than ‘-24’ as threshold for targeting; **Targeted:** ‘yes’ means miRNA targeting to 3’-UTR including SNP rs136632043, if not symbolized as ‘no’.
